# Eleven-Year Incidence of Salivary Gland Tumors—A Retrospective, Single-Centered Study in Croatia

**DOI:** 10.3390/clinpract15060104

**Published:** 2025-05-29

**Authors:** Anđela Modrić, Mirko Gabelica, Ante Mihovilović, Stipe Dumančić, Ana Dunatov Huljev, Ivana Medvedec Mikić

**Affiliations:** 1 Private Dental Office, Put Murvice 12C, 23000 Zadar, Croatia; andela.modric@gmail.com; 2 Department of Otorhinolaryngology with Head and Neck Surgery, University Hospital Center of Split, Spinciceva 1, 21000 Split, Croatia; mgabelica@kbsplit.hr; 3 Department of Maxillofacial Surgery, University Hospital Center of Split, Spinciceva 1, 21000 Split, Croatia; amihovilovic@kbsplit.hr; 4 Clinic for Women’s Diseases and Deliveries, University Hospital Center of Split, Put iza Nove Bolnice 228, 21000 Split, Croatia; sdumancic@kbsplit.hr; 5 Clinical Department of Pathology, Forensic Medicine and Cytology, University Hospital Center of Split, Spinciceva 1, 21000 Split, Croatia; adunatov@kbsplit.hr; 6 Department of Dental Medicine, University Hospital Center of Split, Spinciceva 1, 21000 Split, Croatia; 7 School of Medicine, University of Split, Soltanska 2A, 21000 Split, Croatia

**Keywords:** salivary glands, incidence, head and neck neoplasms, benign neoplasms, malignant neoplasms

## Abstract

Background/Objectives: The purpose of this study was to determine the incidence of salivary gland tumors based on age, gender, histological type, and localization over an eleven-year period at the University Hospital of Split. Methods: The medical records of the Department of Otorhinolaryngology with Head and Neck Surgery and the Department of Maxillofacial Surgery at the University Hospital of Split regarding salivary gland tumors were searched from January 2012 to December 2022. The current fifth World Health Organization (WHO) Classification of Head and Neck Tumors and its criteria were considered during that process. Results: Out of 404 patients, 211 (52.20%) were female and 193 (47.77%) male. The mean age was 60. There were four pediatric patients. Six patients had a combination of two different histological types of salivary gland tumors present simultaneously at the exact localization. Therefore, there were 410 histological types in total, 214 related to females and 196 to males. A total of 361 (88.05%) benign and 49 (11.95%) malignant primary salivary gland tumors were detected. The parotid gland was the predominant location (*N* = 361, 87.8%). There were no cases affecting the sublingual gland. Pleomorphic adenoma was the most common benign histological type (*N* = 169, 41.2%). The most common malignant histological types were adenoid cystic carcinoma (*N* = 9, 2.2%) and mucoepidermoid carcinoma (*N* = 9, 2.2%). The average incidences of salivary gland tumors in the 11 years for the four Dalmatian counties and the Republic of Croatia were 4.45/100,000 and 0.9/100,000, respectively. Conclusions: The results of this study, primarily the ones concerning histological types and localization, do not deviate from general knowledge about salivary gland tumors. Simultaneous and ipsilateral occurrence of different histological types is a rare and extremely valuable finding. The average incidence for Dalmatian counties and the Republic of Croatia is within the range of the International Agency for Research on Cancer estimates.

## 1. Introduction

Salivary gland tumors are a rare group of neoplasms that constitute around 3–10% of all head and neck tumors, depending on the author, with an incidence range from 0.4 to 13.5 per 100,000 inhabitants on a global level, according to the International Agency for Research on Cancer [1,2,3,4,5]. The incidence of malignant salivary gland tumors commonly ranges from 0.4 to 2.6 per 100,000 inhabitants, represented with variations by geographical area, with an incidence of 1.8 per 100,000 inhabitants found in males in Croatia [4,5,6,7].

The etiology of salivary gland tumors is primarily unknown. Possible causes vary from exposure to irradiation, previous health conditions (UV-related skin tumors, breast cancer, immunosuppression, and Hodgkin), occupational exposure to certain chemicals, and poor lifestyle habits such as smoking or high alcohol intake [8,9,10,11,12,13,14,15]. Chromosomic translocation, chemotherapy, or epigenetic alterations may also be etiological factors for the development of salivary gland tumors [16].

A general rule applies to salivary gland tumors: the smaller the salivary gland, the more likely the tumor is to be malignant. Unlike parotid and submandibular gland tumors, which are predominantly benign, sublingual gland tumors are rare and usually malignant, as are the majority of minor salivary gland tumors [17,18]. The most common site for salivary gland tumors is the parotid gland, followed by minor salivary glands [19].

This study, the first of its kind at the University Hospital Center of Split, Croatia, determined the incidence of salivary gland tumors based on age, gender, histological type, and localization over eleven years. It is a single-centered, retrospective study.

The histological types of tumors originating from salivary glands provide heterogeneity unlike any other anatomical site [20]. Because of the relatively low incidence rates, each new finding is significant. The simultaneous occurrence of different histological types of salivary gland tumors is extremely rare, let alone ipsilateral, with only a handful of publications on this subject [21,22,23,24,25]. This makes our finding of six patients with a combination of two different histological types of salivary gland tumors present simultaneously at the exact localization all the more important.

The classification of salivary gland tumors has undergone many changes since the first WHO classification in 1972. The list of benign and malignant tumors and other salivary gland entities has been expanded and re-classified over the years, leading to a better understanding of the subject and targeted therapy approaches. The current fifth WHO classification introduces new entities and eliminates or re-classifies some existing ones, which inevitably changes the structure and incidence rates of specific histological types [26].

## 2. Materials and Methods

**Figure 1:**
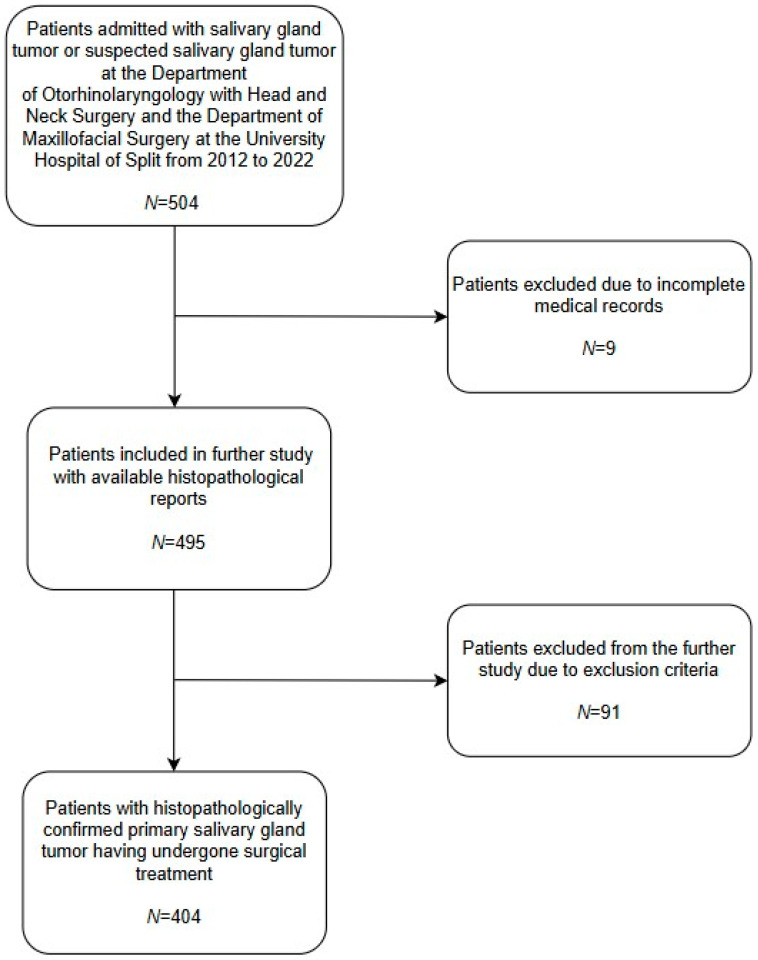
Patient inclusion flow-chart. Out of the total number of patients (*N* = 504) admitted to the University Hospital of Split, nine (*N* = 9) patients were excluded due to incomplete medical records. An additional ninety-one (*N* = 91) patients were excluded due to the following criteria: presence of metastatic salivary gland tumors (e.g., metastatic SCC, metastatic BCC, or metastatic melanoma) or any histopathological diagnosis other than primary salivary gland tumor (e.g., Hodgkin/non-Hodgkin lymphoma, chronic sialadenitis, cyst, lipoma, hemangioma, etc.). The final number of patients with primary salivary gland tumors was four hundred and four (*N* = 404).

Researchers at the University Hospital Center of Split conducted the study at the Department of Otorhinolaryngology with Head and Neck Surgery and the Department of Maxillofacial Surgery. The medical records of both departments regarding salivary gland tumors, from 1 January 2012 to 31 December 2022, were searched. During the reviewed period, the University Hospital Center of Split admitted 504 patients with or with suspected salivary gland tumors. After reviewing patients’ medical records, nine patients had incomplete medical records with no histopathological report available to researchers, as shown in Figure 1, and were excluded. An additional ninety-one (*N* = 91) patients were excluded due to the following criteria: presence of metastatic salivary gland tumors (e.g., metastatic squamous cell carcinoma (SCC), metastatic basal cell carcinoma (BCC), or metastatic melanoma) or any histopathological diagnosis other than primary salivary gland tumor (e.g., Hodgkin/non-Hodgkin lymphoma, chronic sialadenitis, cyst, lipoma, hemangioma, etc.). All histological types taken into account are based on the current fifth WHO Classification of Head and Neck Tumors. The final number of patients with primary salivary gland tumors undergoing surgical treatment was 404. Researchers obtained age, gender, histological type, and tumor localization from the medical records of selected patients, which were further analyzed.

Researchers presented the incidence of benign and malignant salivary gland tumors both as cumulative and annually between 2012 and 2022. Given the large geographical area served by the University Hospital Center of Split, as the largest medical institution in the Dalmatia Region (approximately ¼ of the entire population of Republic of Croatia), the authors presented the incidence relative to the combined population of four counties in the Dalmatia Region and the total population of Croatia They calculated the incidence using population estimates from the Croatian Bureau of Statistics for each year of the study and data from the 2021 Census of population, households, and apartments in Croatia.

The authors used Microsoft Excel from Microsoft 365 MSO (Microsoft Corporation, Redmond, Washington, DC, USA) for data registration and conducted a statistical analysis using JASP software (V. 0.17.3) 2023 (JASP Team, Amsterdam, The Netherlands). Data were tested for the normality of distribution using the Kolmogorov–Smirnov test. Researchers described continuous variables using measures of central tendency (median, minimum, and maximum) and categorical variables using number (*N*) and frequency (%). Differences in continuous variables between study groups were tested with the Mann–Whitney U test or chi-squared test according to the variable type and data distribution. In contrast, the differences in categorical variables were tested using the chi-square test or Fisher’s exact test in the case of small samples or when data was sparse for any histological type of salivary gland tumor. The authors considered a significance level of 0.05 (*p* < 0.05) statistically significant.

The study was conducted following the Declaration of Helsinki, and the protocol was approved by the Ethics Committee of the University Hospital Center of Split (Class: 500-03/22-01/220, No: 2181-147/01/06/LJ.Z.-22-02, from 29 December 2022). At the time of admission to the hospital and before the surgical procedure of tumor removal, all patients signed informed consent, which included the sentence that they agree with the use of their data for possible scientific publications.

## 3. Results

During the 11 years, the University Hospital Center of Split treated 404 patients with primary salivary gland tumors. Most were female patients (*N* = 211, 52.20%), while 193 cases (47.77%) were male patients.

Out of 404 patients, six of them had a combination of two different histological types of salivary gland tumors present simultaneously at the exact localization. Therefore, the authors analyzed the total number of histological types of salivary gland tumors (*N* = 410) rather than the number of patients. Those related to females were 214 cases, and 196 occurred in the male gender. These combinations of tumors were present in the parotid gland as follows: one patient with a benign and malignant tumor (Warthin’s tumor and mucoepidermoid carcinoma), one patient with two malignant tumors (adenoid cystic carcinoma and mucoepidermoid carcinoma), and four patients with two benign tumors (two patients with pleomorphic adenoma and Warthin’s tumor, one with oncocytoma and Warthin’s tumor, and one with basal cell adenoma and Warthin’s tumor).

**Figure 2:**
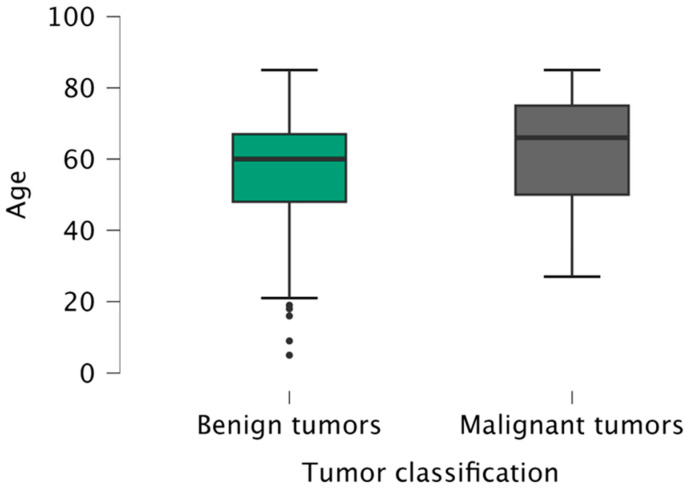
Age distribution of patients according to tumor classification (benign/malignant).

The mean age of the patients was 60 years (5–85 years), slightly higher for males (61 years versus 59 years for females). The mean age of patients with benign tumors (60 years) was statistically significantly lower (*p* = 0.033) than the patients with malignant tumors (66 years), regardless of gender or localization (Figure 2).

The study included four pediatric patients, three girls and one boy, aged 5 to 18. The age distribution by decade was equal, with two pediatric patients in the first and two in the second decade.

Using the chi-squared test, we found statistically significant differences in gender distribution according to tumor localization (*p* = 0.002), with parotid gland tumors being more common in males and submandibular gland and minor salivary gland tumors more common in females. There was no significant difference in gender distribution according to tumor classification.

When observing the histological types of salivary gland tumors according to the age and gender of the patients, a significantly higher proportion of females had pleomorphic adenoma. Conversely, Warthin’s tumor and squamous cell carcinoma were more frequent in males. Both benign and malignant tumors showed a significant difference in occurrence according to gender, with benign tumors seen more often in females (Table 1).

As shown in Table 2, the only statistically significant age difference between patients with different histological types of tumors was noted in female patients who were older than males diagnosed with adenoid cystic carcinoma.

Data showed 361 (88.05%) benign and 49 (11.95%) malignant tumors in the salivary glands, as seen in Table 3. The parotid gland was the predominant location. There were no cases of primary salivary gland tumors affecting the sublingual gland. Differences in the proportion of patients according to the localization and tumor classification were tested with a chi-squared test and found to be statistically significant (*p* < 0.001).

Table 3 shows the distribution of histological types of tumors according to localization, and Table 4 shows the distribution of histological types in individual minor salivary glands.

All of the pediatric patients had pleomorphic adenoma of the parotid gland.

Logistic regression showed that the male gender presents a risk factor for the occurrence of parotid gland tumors (*p* < 0.001). In contrast, submandibular gland tumors (*p* = 0.012) and salivary gland tumors in general (*p* = 0.032) are less common in males. Age has no effect.

Putting the effects of localization, age, and gender on the occurrence of benign or malignant tumors in the prediction model shows that the submandibular gland (*p* < 0.001) and minor salivary glands (*p* < 0.001) are the more frequent locations for the occurrence of malignant tumors. Age and gender do not affect tumor localization significantly.

**Figure 3:**
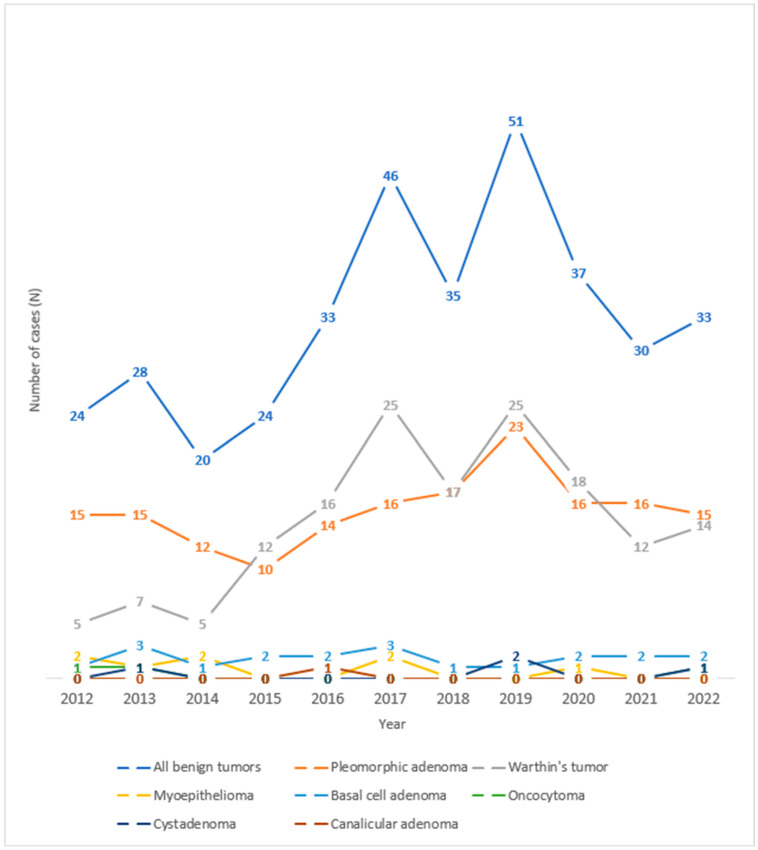
Trend lines for histological types of benign salivary gland tumors operated at the University Hospital of Split throughout the observed years.

Observing the trend of salivary gland tumors throughout the years under review at the University Hospital Center of Split, one can observe an increase in the number of benign salivary gland tumors in 2017 and 2019 (Figure 3). Looking at the histological types of benign tumors of the salivary glands over the observed years, one can see an increase in the number of cases of Warthin’s tumor in 2017 and 2019 and pleomorphic adenoma in 2019.

**Figure 4:**
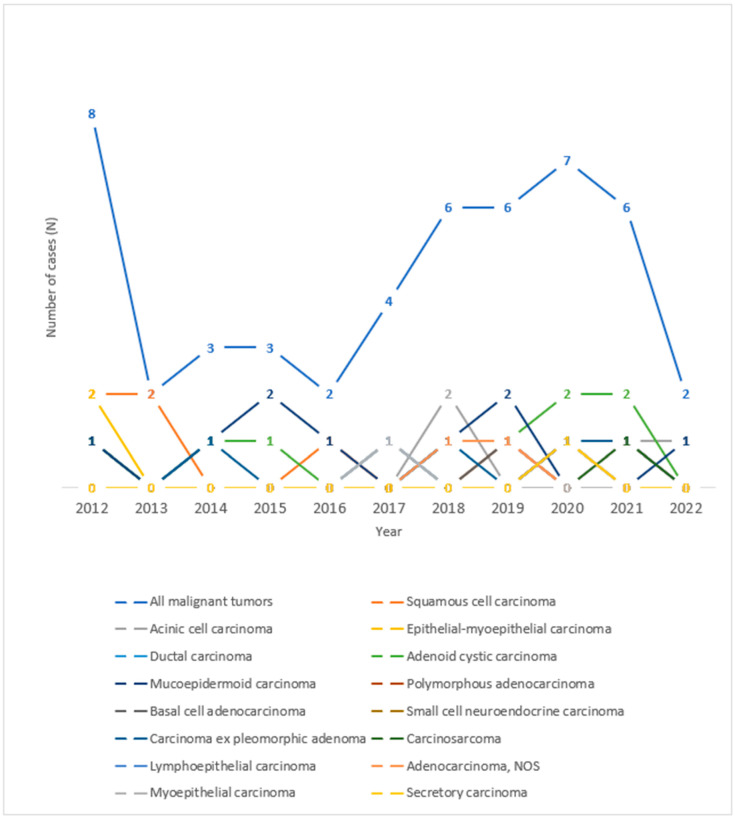
Trend lines for histological types of malignant salivary gland tumors operated at the University Hospital of Split throughout the observed years.

As for the histological types of malignant tumors of the salivary glands, in the period observed, the highest number of tumors was recorded in the years 2012 and 2020, as can be seen in Figure 4. An increase in the number of cases of mucoepidermoid carcinoma in 2015 and 2019, acinic cell carcinoma in 2018, and adenoid cystic carcinoma in 2020 and 2021 can be seen throughout the observed years.

As mentioned (see Materials and Methods), the residents of four surrounding counties gravitate towards the University Hospital Center of Split. Therefore, we were interested in the incidence of benign and malignant tumors per 100,000 inhabitants of the four Dalmatian counties and the total population in Croatia for the examined years. The average incidences of all salivary gland tumors in the 11 years for the Dalmatian counties and the Republic of Croatia were 4.45/100,000 and 0.9/100,000, respectively. The incidence of benign salivary gland tumors for the counties was 3.9/100,000, and was 0.8/100,000 for the population of Croatia. As for malignant tumors, the incidence was 0.53/100,000 for the counties and 0.1/100,000 for Croatia. Table 5 presents the results for individual years.

## 4. Discussion

Salivary gland tumors are histologically the most diverse and sporadic group of tumors. Therefore, the studies regarding this topic are of great value for the insight into the incidence and demographic structure of particular histological types of tumors. This study is the first of its kind conducted at the University Hospital Center of Split. To our knowledge, the only other available data about this specific topic in the Republic of Croatia was published in 2012 by Lukšić et al. [20].

The data from this study showed that salivary gland tumors were more common in female patients than males, following the results of several studies [27,28], as opposed to the data presented in the survey by Lukšić et al. [20]. Comparing the gender and tumor localization, submandibular gland tumors and minor salivary gland tumors were more common in female patients [20]. Additionally, females had pleomorphic adenoma more often than male patients (1.7:1).

In the Chilean study, females accounted for 60.7% of pleomorphic adenoma cases, and researchers reported similar results [28]. Additionally, in a Danish national survey on parotid gland tumors, women accounted for 63.1% of all pleomorphic adenomas [29].

The mean age of patients in our study was 60. The youngest patient was five years old at the moment of surgery, and the eldest was 85. The mean age of patients with benign tumors was significantly higher than that of those with malignant tumors, regardless of gender or localization. The same findings were published in studies by Araya et al. and Lukšić et al. [20,28].

As for pediatric patients in our study, pleomorphic adenoma of the parotid gland was the only entity found. Studies on salivary gland tumors in the pediatric population are rare, with only a few extensive studies published. Results of the study conducted at the Memorial Sloan Kettering Cancer Center in New York on the epithelial neoplasms in the pediatric population showed that the parotid gland was the most common site for salivary gland tumors. In 33% of cases, it was pleomorphic adenoma, while the remaining 67% of cases were malignant tumors [30].

Benign tumors constituted the majority of the cases in this study, approximately 88%. This percentage was significantly higher than that presented in research by Gontarz et al., where benign tumors accounted for approximately 70% of cases [27]. Numerous other studies in which benign tumors prevailed show data similar to Gontarz et al. [20,28].

Based on the results of this study, the parotid gland was the most common site for salivary gland tumors, followed by the submandibular gland and minor salivary glands. There were no cases affecting the sublingual gland. In their study, Aray et al. reported no sublingual gland tumors. As for the minor salivary glands, the benign/malign ratio was identical to this study’s (1:1) [28].

Of the 410 histological types of salivary gland tumors, pleomorphic adenoma was the most common benign salivary gland tumor, followed by Warthin’s, basal cell adenoma, and myoepithelioma. Gonzalez et al. [27] published the same results. A study conducted in Italy on a small patient sample found Warthin’s tumor as the most common histological type, in 55.1% of cases [31].

When we analyzed the frequency of a particular histological type according to localization, we found Warthin’s tumor to be the most common histological type in the parotid gland, and pleomorphic adenoma in the submandibular gland and minor salivary glands. This is contrary to the results of numerous studies that describe pleomorphic adenoma not only as the most common histological type in general but also as the most common histological type in the parotid gland [20,27,28,32]. Nearly all cases of basal cell adenoma and myoepithelioma in this study were found in the parotid gland, following the results of several other studies [20,27,28,33].

Adenoid cystic carcinoma and mucoepidermoid carcinoma were the most common malignant histological types of salivary gland tumors in our study. A Danish national survey published in 2020 indicated adenoid cystic carcinoma as the leading malignant histological type of salivary gland tumors, followed by mucoepidermoid carcinoma [34]. Mucoepidermoid carcinoma, adenoid cystic carcinoma, and acinic cell carcinoma were the three most common histological types in the research by Koivunen et al. [35]. Primary squamous cell carcinoma of the salivary gland is sporadic. However, in our study, it was the third most common malignant histological type. The high frequency of this type of tumor was also noted in research by Westergaard-Nielsen et al. [34].

Analyzing the most common localization of particular histological types of malign salivary gland tumors, the prevailing histological type of tumor in the submandibular gland was adenoid cystic carcinoma, with 50% of all cases, which is similar to the results of a study by Lukšić et al. and a Danish national study [20,34]. As for the parotid gland, the three most common histological types of malignant tumors were primary squamous cell carcinoma, acinic cell carcinoma, and mucoepidermoid carcinoma.

Out of 16 tumors of the minor salivary glands in this study, there were an equal number of benign and malignant tumors. The most common site was the palate, followed by the buccal mucosa, similar to the results of a study published by Araya et al. [28]. The three most common histological types of minor salivary gland tumors in our study were pleomorphic adenoma, mucoepidermoid carcinoma, and adenoid cystic carcinoma, the same histological types found by Lukšić et al. in their research [20].

Most available studies on salivary gland tumors presented one histological type of tumor per patient. Simultaneous occurrence of different histological types of salivary gland tumors is extremely rare, let alone ipsilateral, with only a handful of publications on this subject [21,22,23,24,25]. Of the 404 patients in this study, six had a combination of two different histological types. Lu et al. reported results consistent with our research, with mucoepidermoid carcinoma and Warthin’s tumor being the most common combination. In contrast, the most common individual histological type in such cases was Warthin’s tumor [36].

With minor oscillations, we note an increase in the incidence of benign tumors since 2016, with a peak value in 2019. The incidence of malignant tumors peaked in 2012, and then in 2022, and after a few stable years, the authors noticed a significant decrease. A study conducted on the population of Nottingham over 20 years found the incidence of salivary gland tumors to be significantly higher than in our research: 7.03–8.58/100,000 for all salivary gland tumors, including 6.2–7.2 for benign and 0.83–1.38 for malignant tumors [32]. Gonzalez et al. noticed an increase in incidence in their study over 26 years [27]. Authors of a Danish national survey on malignant tumors of salivary glands over 20 years noted a similar incidence of malignant tumors, with an increase of 1.5% between two decades [34]. The average incidence of salivary gland tumors in our study for the Dalmatian counties and the Republic of Croatia was within the range cited in the published literature (ranging from 0.4 to 13.5 per 100,000) [1,2,3,4,5].

This study did not reflect on the environmental factors. At the time of data collection, researchers did not have access to information regarding patient occupational exposure or lifestyle habits. Having insight into the potential etiology of salivary gland tumors would be a great addition to this study. Besides the information gathered through anamnesis, other potential investigations could be done more objectively, some which would be relatively easy to conduct. Such an example is a study on serum levels of certain trace elements that could be linked to the development of salivary gland tumors and potential malignancies in other sites [37,38]. The advantage of such an approach is that it could be conducted in another medical institution, and the conjoint results could be published as a multi-center study.

## 5. Conclusions

The results of this single-center study, primarily the ones concerning histological types and localization, do not deviate from general knowledge about salivary gland tumors. Missing data from nine patients excluded from this research can be considered a limitation of this study, as these potential data could change the number of cases and statistics altogether. The high frequency of otherwise sporadic primary squamous cell carcinoma and six cases of extremely rare ipsilateral simultaneous occurrence of two different histological types of tumor could be considered our most valuable findings. The incidence of salivary gland tumors in general, especially the malignant histological types, was higher among the population of four Dalmatian counties than that based on the population of Croatia. These data present a valuable contribution to general knowledge on salivary gland tumors; surely, future research on this topic is needed.

## Figures and Tables

**Table 1:** Gender distribution according to histological types of salivary gland tumors presented as number of cases and percentage, *N* (%).

Histological Type	Occurrence ^†^	Gender		*p*-Value
		Female	Male	
**Benign tumors**				
Pleomorphic adenoma	169 (41.2)	107 (63.3)	62 (36.7)	<0.001 ^a^*
Warthin’s tumor	156 (38)	60 (38.5)	96 (61.5)	<0.001 ^a^*
Myoepithelioma	8 (2)	5 (62.5)	3 (37.5)	0.726 ^b^
Basal cell adenoma	20 (4.9)	14 (70)	6 (30)	0.113 ^b^
Oncocytoma	3 (0.7)	0	3 (100)	0.108 ^b^
Cystadenoma	4 (1)	0	4 (100)	0.051 ^b^
Canalicular adenoma	1 (0.2)	1 (100)	0	1.0 ^b^
All benign tumors	361	188 (52.1)	173 (47.9)	<0.001 ^a^*
**Malignant tumors**				
Squamous cell carcinoma	7 (1.7)	0	7 (100)	0.005 ^b^*
Acinic cell carcinoma	5 (1.2)	5 (100)	0	0.062 ^b^
Epithelial–myoepithelial carcinoma	3 (0.7)	1 (33.3)	2 (66.7)	0.608 ^b^
Ductal carcinoma	3 (0.7)	1 (33.3)	2 (66.7)	0.608 ^b^
Adenoid cystic carcinoma	9 (2.2)	5 (55.6)	4 (44.4)	1.0 ^b^
Mucoepidermoid carcinoma	9 (2.2)	7 (77.8)	2 (22.2)	0.179 ^b^
Polymorphous adenocarcinoma	1 (0.2)	1 (100)	0	1.0 ^b^
Basal cell adenocarcinoma	1 (0.2)	0	1 (100)	0.478 ^b^
Small cell neuroendocrine carcinoma	1 (0.2)	0	1 (100)	0.478 ^b^
Carcinoma ex pleomorphic adenoma	4 (1)	1 (25)	3 (75)	0.353 ^b^
Carcinosarcoma	1 (0.2)	1 (100)	0	1.0 ^b^
Lymphoepithelial carcinoma	1 (0.2)	1 (100)	0	1.0 ^b^
Adenocarcinoma, NOS	2 (0.5)	2 (100)	0	0.500 ^b^
Myoepithelial carcinoma	1 (0.2)	0	1 (100)	0.478 ^b^
Secretory carcinoma	1 (0.2)	1 (100)	0	1.0 ^b^
All malignant tumors	49	26 (53.1)	23 (46.9)	0.044 ^b^*

* Comparisons of gender distribution according to the histological type of salivary gland tumors was tested with chi-squared (*X*^2^) ^a^ or Fisher’s exact test ^b^, *p* < 0.05. ^†^ On a total sample of 404 patients in this study, six patients had a combination of two different histological types of salivary gland tumors present simultaneously at the same localization, which led to a number of 410 histological types of tumors on which analysis was conducted. Occurrence is presented concerning the total number of histological types (*N* = 410).

**Table 2:** The age distribution according to histological types of salivary gland tumors. Occurrence is presented as number of cases and percentage, *N* (%). Age is presented as median (min–max), with CI.

Histological Type	Occurrence ^†^	Age		*p*-Value
		Female	Male	
**Benign tumors**				
Pleomorphic adenoma	169 (41.2)	55 (5–84) CI (48.7–55.5)	53.5 (9–82) CI (47.9–53.5)	0.965
Warthin’s tumor	156 (38)	62 (30–84) CI (59.2–62)	63 (31–85) CI (59.6–63.4)	0.857
Myoepithelioma	8 (2)	42 (36–84) CI (25.3–74.7)	49 (37–52) CI (26.2–65.7)	0.881
Basal cell adenoma	20 (4.9)	60 (33–76) CI (52.2–67.4)	59.5 (33–81) CI (41.8–78.9)	0.869
Oncocytoma	3 (0.7)	0	70 (59–73) CI (49–85.6)	Na
Cystadenoma	4 (1)	0	55.5 (49–85) CI (35.2–87.3)	Na
Canalicular adenoma	1 (0.2)	0	0	Na
All benign tumors	361	59 (5–84) CI (53.1–57.7)	60 (9–85) CI (55.9–61.2)	0.221
**Malignant tumors**				
Squamous cell carcinoma	7 (1.7)	0	80 (43–82) CI (59.3–85.3)	Na
Acinic cell carcinoma	5 (1.2)	50 (27–79) CI (20.5–82.7)	0	Na
Epithelial–myoepithelial carcinoma	3 (0.7)	0	39.5 (29–50) CI (22.7–43.8)	Na
Ductal carcinoma	3 (0.7)	0	76 (75–77) CI (73.9–86.2)	Na
Adenoid cystic carcinoma	9 (2.2)	74 (69–79) CI (68.8–78.4)	55.5 (29–65) CI (26.6–75.9)	0.016 *
Mucoepidermoid carcinoma	9 (2.2)	54 (36–79) CI (43.5–67.6)	70 (65–75) CI (6.5–133.5)	0.184
Polymorphous adenocarcinoma	1 (0.2)	0	0	Na
Basal cell adenocarcinoma	1 (0.2)	0	0	Na
Small cell neuroendocrine carcinoma	1 (0.2)	0	0	Na
Carcinoma ex pleomorphic adenoma	4 (1)	0	46 (39–82) CI (34.8–50.1)	Na
Carcinosarcoma	1 (0.2)	0	0	Na
Lymphoepithelial carcinoma	1 (0.2)	0	0	Na
Adenocarcinoma, NOS	2 (0.5)	48 (29–67) CI (19.2–56.3)	0	Na
Myoepithelial carcinoma	1 (0.2)	0	0	Na
Secretory carcinoma	1 (0.2)	0	0	Na
All malignant tumors	49	59 (28–85) CI (51.6–66.7)	70 (29–82) CI (55.5–71.7)	0.283

*Na* not available. * Comparison of age between genders, according to histological type of salivary gland tumor, was tested with the Mann–Whitney U test, *p* < 0.05. ^†^ On a total sample of 404 patients in this study, six patients had a combination of two different histological types of salivary gland tumors present simultaneously at the exact localization, so 410 histological types of tumors were analyzed. Occurrence is presented concerning the total number of histological types (*N* = 410).

**Table 3:** Histological types of salivary gland tumors according to localization, presented as number of cases and percentage, *N* (%).

Histological Type	Parotid Gland	Submandibular Gland	Sublingual Gland	Minor Salivary Glands
Benign tumors				
Warthin’s tumor	155 (47.0)	1 (4.4)	0	0
Pleomorphic adenoma	144 (43.6)	20 (86.9)	0	5 (62.5)
Basal cell adenoma	17 (5.7)	1 (4.4)	0	2 (25)
Myoepithelioma	6 (1.8)	1 (4.4)	0	1 (12.5)
Cystadenoma	4 (1.2)	0	0	0
Oncocytoma	3 (0.9)	0	0	0
Canalicular adenoma	1 (0.3)	0	0	0
All benign tumors	330	23	0	8
Malignant tumors				
Squamous cell carcinoma	5 (16.1)	2 (20)	0	0
Acinic cell carcinoma	5 (16.1)	0	0	0
Mucoepidermoid carcinoma	5 (16.1)	1 (10)	0	3 (37.5)
Carcinoma ex pleomorphic adenoma	4 (12.9)	0	0	0
Ductal carcinoma	3 (9.7)	0	0	
Epithelial–myoepithelial carcinoma	1 (3.2)	2 (20)	0	0
Adenoid cystic carcinoma	1 (3.2)	5 (50)	0	3 (37.5)
Polymorphous adenocarcinoma	1 (3.2)	0	0	0
Basal cell adenocarcinoma	1 (3.2)	0	0	0
Small cell neuroendocrine carcinoma	1 (3.2)	0	0	0
Carcinosarcoma	1 (3.2)	0	0	0
Lymphoepithelial carcinoma	1 (3.2)	0	0	0
Adenocarcinoma, NOS	1 (3.2)	0	0	1 (12.5)
Secretory carcinoma	1 (3.2)	0	0	0
Myoepithelial carcinoma	0	0	0	1 (12.5)
All malignant tumors	31	10	0	8
Total	361	33	0	16

**Table 4:** Distribution of histological types of tumors according to localization in individual minor salivary glands, presented as number of cases and percentage, *N* (%).

Histological Type	Minor Salivary Glands				
	Palate	Floor of the Mouth	Buccal Mucosa	Lip	Total
**Benign tumors**					
Warthin’s tumor	0	0	0	0	0
Pleomorphic adenoma	5 (71.4)	0	0	0	5 (62.5)
Basal cell adenoma	1 (14.3)	0	1 (100)	0	2 (25)
Myoepithelioma	1 (14.3)	0	0	0	1 (12.5)
Cystadenoma	0	0	0	0	0
Oncocytoma	0	0	0	0	0
Canalicular adenoma	0	0	0	0	0
All benign tumors	7	0	1	0	8
**Malignant tumors**					
Squamous cell carcinoma	0	0	0	0	0
Acinic cell carcinoma	0	0	0	0	0
Mucoepidermoid carcinoma	2 (40)	1 (100)	0	0	3 (37.5)
Carcinoma ex pleomorphic adenoma	0	0	0	0	0
Ductal carcinoma	0	0	0	0	
Epithelial–myoepithelial carcinoma	0	0	0	0	0
Adenoid cystic carcinoma	2 (40)	0	1 (100)	0	3 (37.5)
Polymorphous adenocarcinoma	0	0	0	0	0
Basal cell adenocarcinoma	0	0	0	0	0
Small cell neuroendocrine carcinoma	0	0	0	0	0
Carcinosarcoma	0	0	0	0	0
Lymphoepithelial carcinoma	0	0	0	0	0
Adenocarcinoma, NOS	0	0	0	1 (100)	1 (12.5)
Secretory carcinoma	0	0	0	0	0
Myoepithelial carcinoma	1 (20)	0	0	0	1 (12.5)
All malignant tumors	5	1	1	1	8
**Total**	12	1	2	1	16

**Table 5:** Incidence of benign and malignant salivary gland tumors in the examined years on a sample of the total population of four Dalmatian counties and the population of the Republic of Croatia. Values are presented as an absolute number per 100,000 inhabitants.

Year	Population Sample			
	Dalmatian Counties		Republic of Croatia	
	Benign Tumors	Malignant Tumors	Benign Tumors	Malignant Tumors
**2012**	2.80	0.94	0.56	0.19
**2013**	3.28	0.23	0.66	0.05
**2014**	2.34	0.35	0.47	0.07
**2015**	2.82	0.35	0.57	0.07
**2016**	3.90	0.24	0.79	0.05
**2017**	5.47	0.48	1.12	0.1
**2018**	4.18	0.72	0.86	0.15
**2019**	6.09	0.72	1.26	0.15
**2020**	4.42	0.84	0.91	0.17
**2021**	3.77	0.76	0.78	0.15
**2022**	4.15	0.25	0.86	0.05

## Data Availability

The authors will make the raw data supporting this article’s conclusions available upon request.

## Abbreviations

The following abbreviations are used in this manuscript:

WHO	World Health Organization
SCC	Squamous cell carcinoma
BCC	Basal cell carcinoma

## References

[1] Stryjewska-Makuch G., Kolebacz B., Janik M.A., Wolnik A. (2017). Increase in the incidence of parotid gland tumors in the years 2005-2014. Otolaryngol. Pol..

[2] Chan W.H., Lee K.W., Chiang F.Y., Ho K.Y., Chai C.Y., Kuo W.R. (2010). Features of parotid gland diseases and surgical results in southern Taiwan. Kaohsiung J. Med. Sci..

[3] Fonseca F.P., Carvalho Mde V., de Almeida O.P., Rangel A.L., Takizawa M.C., Bueno A.G., Vargas P.A. (2012). Clinicopathologic analysis of 493 cases of salivary gland tumors in a Southern Brazilian population. Oral Surg. Oral Med. Oral Pathol. Oral Radiol..

[4] Vargas P.A., Gerhard R., Araújo Filho V.J., de Castro I.V. (2002). Salivary gland tumors in a Brazilian population: A retrospective study of 124 cases. Rev. Hosp. Clin..

[5] Barnes L., Eveson J.W., Reichart P.A., Sidranskiy D. (2005). World Health Organization Classification of Tumours. Pathology and Genetics of Head and Neck Tumours.

[6] Parkin D.M., Whelan S.L., Ferlay J., Teppo L., Thomas D.B. (2002). Cancer Incidence in Five Continents, Vol. VIII. IARC Scientific Publications No.155.

[7] Kordzińska-Cisek I., Grzybowska-Szatkowska L. (2018). Salivary gland cancer —Epidemiology. Nowotw. J. Oncol..

[8] Guzzo M., Locati L.D., Prott F.J., Gatta G., McGurk M., Licitra L. (2010). Major and minor salivary gland tumors. Crit. Rev. Oncol. Hematol..

[9] Patel D.K., Morton R.P. (2016). Demographics of benign parotid tumors: Warthin’s tumor versus other benign salivary tumors. Acta Otolaryngol..

[10] Sawabe M., Ito H., Takahara T., Oze I., Kawakita D., Yatabe Y., Hasegawa Y., Murakami S., Matsuo K. (2018). Heterogeneous impact of smoking on major salivary gland cancer according to histopathological subtype: A case-control study. Cancer.

[11] Gatta G., Guzzo M., Locati L.D., McGurk M., Prott F.J. (2020). Major and minor salivary gland tumors. Crit. Rev. Oncol. Hematol..

[12] Cantù G. (2021). Adenoid cystic carcinoma. An indolent but aggressive tumor. Part A: From aetiopathogenesis to diagnosis. Acta Otorhinolaryngol. Ital. Organo Uff. Della Soc. Ital. Otorinolaringol. E Chir. Cerv-Facc..

[13] Swanson G.M., Burns P.B. (1997). Cancers of the salivary gland: Workplace risks among women and men. Ann. Epidemiol..

[14] Forrest J., Campbell P., Kreiger N., Sloan M. (2008). Salivary gland cancer: An exploratory analysis of dietary factors. Nutr. Cancer.

[15] Horn-Ross P.L., Ljung B.M., Morrow M. (1997). Environmental factors and the risk of salivary gland cancer. Epidemiology.

[16] Dos Santos E.S., Ramos J.C., Normando A.G.C., Mariano F.V., Paes Leme A.F. (2020). Epigenetic alterations in salivary gland tumors. Oral Dis..

[17] Walvekar R., Phalke N.P. (2021). The Evaluation and Management of Carcinoma of the Minor Salivary Glands. Otolaryngol. Clin. N. Am..

[18] Park M., Cho J., Ryu J., Jeong H.S. (2021). Diagnosis and management of malignant sublingual gland tumors: A narrative review. Gland Surg..

[19] Dalgic A., Karakoc O., Aydin U., Hidir Y., Gamsizkan M., Karahatay S., Gerek M. (2014). Minor salivary gland neoplasms. J. Craniofacial Surg..

[20] Lukšić I., Virag M., Manojlović S., Macan D. (2012). Salivary gland tumors: 25 years of experience from a single institution in Croatia. J. Cranio-Maxillofac. Surg..

[21] Val-Bernal J.F., Mayorga M.M., Martín-Soler P., Obeso S., Alonso-Fernández E.M., López-Rasines G. (2019). Synchronous Warthin Tumor and Papillary Oncocytic Cystadenoma in the Ipsilateral Parotid Gland: An Unreported Association. Rom. J. Morphol. Embryol..

[22] Klamminger G.G., Issing C., Burck I., Herr C., Endemann E., Stöver T., Wild P.J., Winkelmann R. (2023). Uncommon Coexistence of Pleomorphic Adenoma and Warthin’s Tumor in a Painfully Swollen Left Parotid Gland: A Surgical Case Report. Am. J. Case Rep..

[23] Araki Y., Sakaguchi R. (2004). Synchronous oncocytoma and Warthin’s tumor in the ipsilateral parotid gland. Auris Nasus Larynx.

[24] Stavrianos S.D., McLean N.R., Soames J.V. (1999). Synchronous unilateral parotid neoplasms of different histological types. Eur. J. Surg. Oncol..

[25] Gnepp D.R., Schroeder W., Heffner D. (1989). Synchronous tumors arising in a single major salivary gland. Cancer.

[26] Żurek M., Fus Ł., Niemczyk K., Rzepakowska A. (2023). Salivary gland pathologies: Evolution in classification and association with unique genetic alterations. Eur. Arch. Otorhinolaryngol..

[27] Gontarz M., Bargiel J., Gąsiorowski K., Marecik T., Szczurowski P., Zapała J., Wyszyńska-Pawelec G. (2021). Epidemiology of primary epithelial salivary gland tumors in Southern Poland—A 26-year, clinicopathologic, retrospective analysis. J. Clin. Med..

[28] Araya J., Martinez R., Niklander S., Marshall M., Esguep A. (2015). Incidence and prevalence of salivary gland tumors in Valparaiso, Chile. Med. Oral Patol. Oral Cir. Bucal..

[29] Andreasen S., Therkildsen M.H., Bjørndal K., Homøe P. (2016). Pleomorphic adenoma of the parotid gland 1985-2010: A Danish nationwide study of incidence, recurrence rate, and malignant transformation. Head Neck.

[30] Xu B., Aneja A., Ghossein R., Katabi N. (2017). Salivary gland epithelial neoplasms in pediatric population: A single-institute experience with a focus on the histologic spectrum and clinical outcome. Hum. Pathol..

[31] Tauro F., Cianfrone F., Ralli M. (2021). Retrospective Study of Salivary Gland Tumor Cases in a Large Italian Public Hospital and Review of the Literature. Clin. Ter..

[32] Bradley P.J., McGurk M. (2013). Incidence of salivary gland neoplasms in a defined UK population. Br. J. Oral Maxillofac. Surg..

[33] Santana B.W., Silva L.P., Serpa M.S., Borges M.d.A., Moura S., Silveira M., Sobral A. (2021). Incidence and profile of benign epithelial tumors of salivary glands from a single center in Northeast of Brazil. Med. Oral Patol. Oral Cir. Bucal..

[34] Westergaard-Nielsen M., Godballe C., Eriksen J.G., Larsen S.R., Kiss K., Agander T., Ulhøi B.P., Charabi B., Klug T.E., Jacobsen H. (2021). Salivary gland carcinoma in Denmark: A national update and follow-up on incidence, histology, and outcome. Eur. Arch. Otorhinolaryngol..

[35] Koivunen P., Suutala L., Schorsch I., Jokinen K., Alho O.P. (2002). Malignant epithelial salivary gland tumors in northern Finland: Incidence and clinical characteristics. Eur. Arch. Otorhinolaryngol..

[36] Lu H., Xu W., Zhu Y., Liu L., Liu S., Yang W. (2019). Simultaneous occurrence of benign and malignant tumors in the ipsilateral parotid gland—Retrospective study. Int. J. Oral Maxillofac. Surg..

[37] Jaafari-Ashkavandi Z., Khademi B., Malekzadeh M., Shahmoradi Z. (2019). Serum Levels of Zinc, Copper and Ferritin in Patients with Salivary Gland Tumors. Asian Pac. J. Cancer Prev..

[38] Lubiński J., Lener M.R., Marciniak W., Pietrzak S., Derkacz R., Cybulski C., Gronwald J., Dębniak T., Jakubowska A., Huzarski T. (2023). Serum Essential Elements and Survival after Cancer Diagnosis. Nutrients.

